# The versatile roles of ADAM8 in cancer cell migration, mechanics, and extracellular matrix remodeling

**DOI:** 10.3389/fcell.2023.1130823

**Published:** 2023-02-23

**Authors:** Claudia Tanja Mierke

**Affiliations:** Faculty of Physics and Earth Science, Biological Physics Division, Peter Debye Institute of Soft Matter Physics, Leipzig University, Leipzig, Germany

**Keywords:** extracellular matrix remodeling, fibronectin, cell mechanics, cancer cells, MMP-9, sheddases, EMT, immune cells

## Abstract

The posttranslational proteolytic cleavage is a unique and irreversible process that governs the function and half-life of numerous proteins. Thereby the role of the family of A disintegrin and metalloproteases (ADAMs) plays a leading part. A member of this family, ADAM8, has gained attention in regulating disorders, such as neurogenerative diseases, immune function and cancer, by attenuating the function of proteins nearby the extracellular membrane leaflet. This process of “ectodomain shedding” can alter the turnover rate of a number of transmembrane proteins that function in cell adhesion and receptor signal transduction. In the past, the major focus of research about ADAMs have been on neurogenerative diseases, such as Alzheimer, however, there seems to be evidence for a connection between ADAM8 and cancer. The role of ADAMs in the field of cancer research has gained recent attention, but it has been not yet been extensively addressed. Thus, this review article highlights the various roles of ADAM8 with particular emphasis on pathological conditions, such as cancer and malignant cancer progression. Here, the shedding function, direct and indirect matrix degradation, effects on cancer cell mobility and transmigration, and the interplay of ADAM8 with matrix-embedded neighboring cells are presented and discussed. Moreover, the most probable mechanical impact of ADAM8 on cancer cells and their matrix environment is addressed and debated. In summary, this review presents recent advances in substrates/ligands and functions of ADAM8 in its new role in cancer and its potential link to cell mechanical properties and discusses matrix mechanics modifying properties. A deeper comprehension of the regulatory mechanisms governing the expression, subcellular localization, and activity of ADAM8 is expected to reveal appropriate drug targets that will permit a more tailored and fine-tuned modification of its proteolytic activity in cancer development and metastasis.

## 1 Introduction

The field of cancer cell research is still only at the tip of the iceberg in terms of the impact of proteolytic degradation. Proteolytic degradation helps to comprehend the entire complexity of tumors and especially their interaction with surrounding tissue. Thus, a focus of cancer research has shifted toward the proteolytic degradation, such as shedding of receptors, and their impact on cancer development and its malignant progression. Moreover, the comparability of *in vitro* studies with *in vivo* model systems has been shown to be rather weak in terms of architecture and dimensionality. In the last decade, this problem has been addressed as there has been a push toward sophisticated 3D *in vitro* cell culture systems that can bridge the existing gap between 2D *in vitro* and *in vivo* cancer research experiments in the field of cell biology and biophysics. In particular, these 3D cell culture model systems made it possible to study the effects of proteolytic degradation, such as a disintegrin and metalloproteinase 8 (ADAM8), and cell mechanical forces on the restructuring of the ECM environment on cancer cell migration. Thereby, the established hypothesis that the bidirectional interaction between cancer cells, such as cancer cells, and their tumor microenvironment is crucial for the prognosis and treatment of the disease has been experimentally tested in 3D *in vitro* cell culture models (Mierke, 2019, 2020, 2021; Baghban et al., 2020; Winkler et al., 2020; Rubenich et al., 2021). The tissue remodeling processes triggered by the cancer cells are particularly relevant for both the mechanical stress exerted by the cancer cells on their matrix environment and the adhesion, migration, and invasion of the cancer cells (Northcott et al., 2018; Voutouri and Stylianopoulos, 2018). Research on remodeling processes has so far focused on matrix metalloproteinases (MMPs) (Rolli et al., 2003; Wolf and Friedl, 2009; Zarrabi et al., 2011; Wolf et al., 2013; Das et al., 2017) rather than members of the ADAM family, suggesting that a major knowledge gap remains to be filled. However, the function of ADAM8 in cell mechanics, which is thought to regulate cellular motility in 3D collagen matrices, remains still largely unclear, but there is some evidence of its importance. In the unprecedented review focusing on ADAM8, it is suggested that the disruption of mechanotransduction may be strongly influenced by ADAM8, as well as the transmission of cellular forces to the cellular environment. The focus of this review is on the relationship between ADAM8 and cancer, with particular emphasis on cancer cell mobility, transendothelial migration of cancer cells, epithelial-to-mesenchymal transition (EMT), and malignant cancer progression such as metastasis. In this review article, the members of the ADAM family, protein structure, ligands/substrates and regulation of ADAM8, as well as the interaction of ADAM8 with other proteins are presented. Special attention is paid to the interaction of ADAM8 with the ECM, which can be both direct and indirect. Finally, the role of ADAM8 in other cells of the ECM, such as macrophages, will also be addressed to complete the picture of the multiple roles of ADAM8.

## 2 ADAM family members and ADAM8

The ADAM family consists of two groups, membrane-anchored ADAM (Table 1) and secreted-type ADAM with thrombospondin motifs (ADAMTS) (Duffy et al., 2009b, 2009a; Weber and Saftig, 2012). In the following, the focus is only on the membrane-anchored and transmembrane ADAMs. The ADAM family members are quite similar in structure to the well-known and widely researched family of MMPs (Wolf and Friedl, 2009; Wolf et al., 2013; Singh et al., 2015). ADAMs are members of the metzincin subset of proteins that comprise the zinc protease superfamily.

**TABLE 1:** Members of the family of ADAMs and their functions with a focus on disorders and diseases.

ADAMs family members	Functionality	Disease	References
ADAM11	Neural development, myelination and synaptic transfer	Epilepsy	
ADAM22		Epilepsy	Bolger and Young (2013). van der Knoop et al. (2022)
		Cancer	
ADAM23	Synapse maturation	Epilepsy	Lovero et al. (2015)
		Esophageal cancer	Chen et al. (2021)
ADAM10	Notch cleavage is needed for development of the nervous system	Cleavage of amyloid precursor protein leads to Alzheimer’s disease	Zhu et al. (2018)
	Selectively regulated by tetraspanins		
ADAM8		Neuroinflammation	Bartsch et al. (2010)
		Gastric cancer, breast cancer, hepatic cancer, lung cancer, pancreas cancer	
ADAM17	Neurite outgrowth, myelination and its activity is regulated by iRhoms	Neuroinflammation	Cui et al. (2015)
	Neural vascular barrier function		
ADAM19	Regenerative processes upon neuronal injury		
ADAM21	Regenerative processes upon neuronal injury		
ADAM9		Pancreatic cancer	
		Alzheimer’s disease	
ADAM15		Alzheimer’s disease	
ADAM30		Alzheimer’s disease	
ADAM12	Neural vascular barrier function		Cui et al. (2015)

In terms of function, ADAMs exhibit two principal biological activities, such as proteolysis and adhesion. These two very distinct activities allow ADAMs to engage in a multitude of diverse activities, encompassing ectodomain shedding from membrane proteins, cell fusion, cell adhesion, cell migration, and cell signal transduction (Duffy et al., 2009b, 2009a; Weber and Saftig, 2012). After their synthesis in the endoplasmic reticulum, the members of the ADAMs family can subsequently mature in the Golgi apparatus prior to travel toward the plasma membrane (Prox et al., 2012; Saraceno et al., 2014). ADAMs are linked to a number of physiological and pathological functions, including cancer and its malignant progression. The best studied of these multiple activities is their role in proteolysis.

There are nearly half of the approximately 34 ADAM family members identified so far in mice and 22 in humans (Zhu et al., 2021) (Table 1) that are specifically or predominantly expressed in a tissue-specific manner, such as in testis or epididymis, suggesting an integral involvement of these ADAMs in reproduction and fertilization in mammals (Weber and Saftig, 2012). However, other ADAMs are expressed in a ubiquitous manner, as they can be found in a broad range of cells and tissues and are implicated in several biological processes, among them development, inflammation, and cancer (Mullooly et al., 2016).

Among the human ADAM family members, only half of them possesses proteolytic activity, comprising ADAM8, ADAM9, ADAM10, ADAM12, ADAM15, ADAM17, ADAM19, ADAM20, ADAM21, ADAM28, and ADAM33 (Table 2), whereas the other half exhibits no proteolytic activity. Among the non-proteolytic members are ADAM11, ADAM22, and ADAM23 that fulfill relevant operations in neural development, myelination and synaptic transmission and are connected with epilepsy (Hivert et al., 2018; Hsia et al., 2019).

**TABLE 2:** ADAMs and their substrates fulfill multiple functions.

ADAMs	Protein state (active/inactive)	Substrate	Functions and possible functions
ADAM8	active	CD23, c-kit ligand (KL-1), TNFα and amyloid precursor protein (APP) Naus et al. (2006)	Proteolytic cleavage of membrane receptors and ECM proteins
		Fibronectin Zack et al. (2009)	Adhesion, angiogenesis, inflammation
		CD30, TNF- a , L1, L-selectin	Tumor progression, invasion, and metastasis (pancreas)
ADAM9	active	Amyloid precursor protein (APP), c-kit ligand (KL-1), collagen XVII, DLL1, EGF, HB-EGF, laminin, TNF- α , ADAM10 (Ectodomain)	Adhesion, angiogenesis, migration, proliferation
ADAM10	active	Amyloid precursor protein (APP), betacellulin, CD23, CD30, CD44, DLL1, E-cadherin, EGF, epigenin, Fas-L, HB-EGF, HER2, L1, N-cadherin, Notch, TNF- α	Adhesion, angiogenesis, cell survival, inflammation, invasion, migration
ADAM12	active	Collagen IV, DLL1, fibronectin, HB-EGF	Angiogenesis, migration, proliferation
ADAM13	active	Amphiregulin, CD23, collagen IV, E-cadherin, HB-EGF, ADAM10 (ectodomain)	Cell-cell adhesion
ADAM17	active	Amphiregulin, amyloid precursor protein (APP), CD44, collagen XVII, DLL1, epiregulin, epigen, HB-EGF, ICAM-1, L-selectin, Notch, TGF- α , TNF- α , VCAM-1, IL6R	Adhesion, angiogenesis, cell survival, inflammation, invasion, migration, proliferation
ADAM19	active	TNF- α , kit-ligand 1 (KL-1), TRANCE	Angiogenesis, adhesion, inflammation, invasion
ADAM28	active	IGBP3, VWF, CD23	Proliferation
ADAM33	active	IL18	Angiogenesis
ADAM1	inactive		Sperm egg binding and fusion
ADAM2	inactive		Sperm egg binding and fusion
ADAM7	inactive		Sperm maturation
ADAM11	inactive		Neural adhesion, integrin ligand
ADAM18	inactive		
ADAM22	inactive		Cell-cell interaction
ADAM23	inactive		Cell-cell interaction
ADAM29	inactive		Cell-cell interaction

The major substrates of ADAM proteases are type I and type II transmembrane proteins, which are single-pass transmembrane proteins, that comprise cytokine and growth factor precursors, growth factor/cytokine receptors, and adhesion proteins (Table 2) (Duffy et al., 2009a; Weber and Saftig, 2012; Jones et al., 2016). The cleavage of most substrates proceeds at a distance of 10–15 amino acids from the plasma membrane.

## 3 Structure, activation, substrates/ligand, and regulation of ADAM8 in comparison to other ADAMs

**FIGURE 1:**
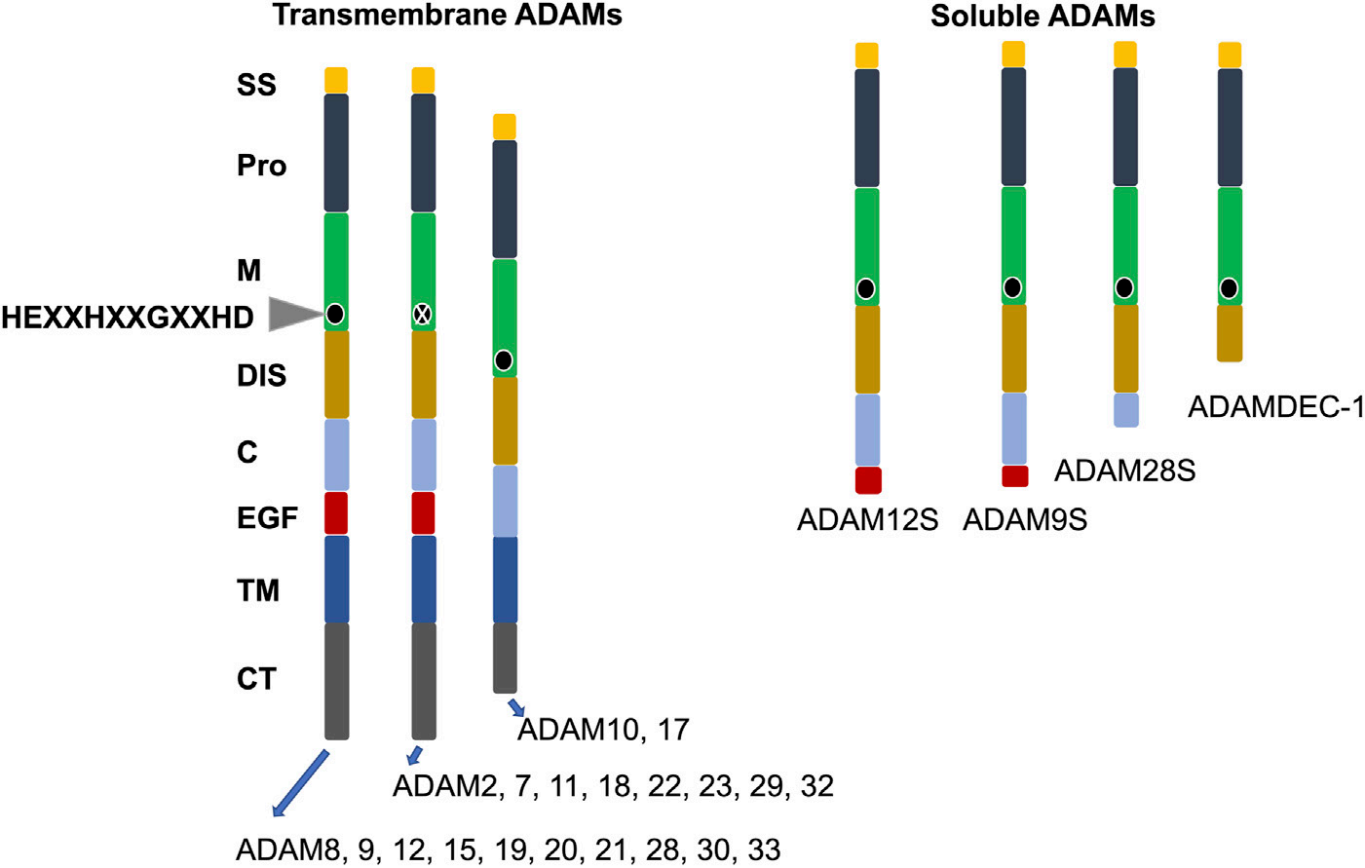
Structural organization of the family of human ADAMs. ADAMs are divided in transmembrane and soluble subgroups. The conserved active site of amino acids is indicated by a black circle. X stands for ADAMs without a conserved active site sequence in their M domain. C, cysteine-rich; CT, cytoplasmic tail; DIS, disintegrin-like; EGF, epidermal growth factor-like; M, metalloproteinase; Pro, prodomain, and SS, signal sequence.

ADAM family proteins exhibit a joint multidomain structure that includes at its N-terminus a signal peptide, thereafter a prodomain (propeptide), which is trailed by metalloprotease, disintegrin, cysteine-rich, transmembrane, and cytoplasmic domains (Figure 1). Several ADAM family members harbor an accessory epidermal growth factor (EGF)-like domain, that is, located intermediately between the cysteine-rich and transmembrane domains. The consensus sequence within the metalloprotease domain is HEXXHXXGXXHD, indicating an active ADAM protease, with histidines binding zinc and glutamic acid, which support the catalytic reaction (Figure 1) (Stöcker et al., 2008). Certain family members of ADAMs, such as ADAM11, ADAM22, and ADAM23, lack the consensus sequence in their metalloprotease domain and are therefore inactive ADAMs participating mainly in direct cell-to-cell signal transduction through attachment to other proteins (Figure 1). The cytoplasmic region of the transmembrane ADAM family members is rather short in comparison to the extracellular domain and harbors tethering sites for diverse intracellular signal transduction proteins (Wolfsberg and White, 1996).

**FIGURE 2:**
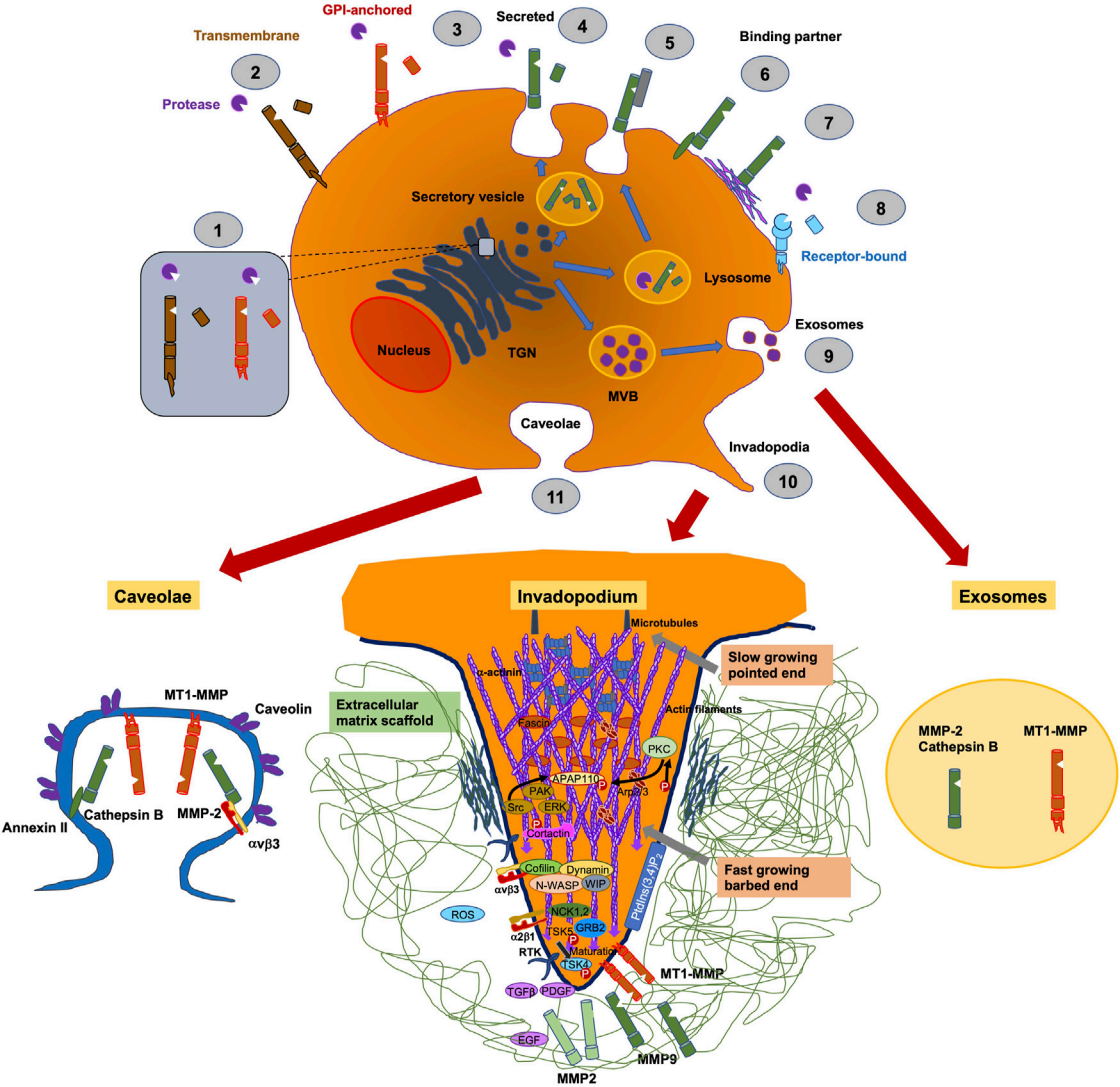
Pericellular proteases, such as ADAM8, are involved in trafficking of proteins. They are synthesized in the ER and get transferred to the Golgi complex until they enter the trans-Golgi-network (TGN). (1) In the TGN membrane, attached proteases are switched active through furin. (2) Membrane-bound proteases are delivered to the cell surface, whereby they are inactive precursor proteins and become active within the perivascular cavity. (3) Traditionally, secreted proteases are transported to the cell membrane *via* a constitutive secretory pathway and activated after their liberation in the pericellular cavity. Proteases in endosomes or lysosomes are transported to the extracellular cavity by different pathways: (4) the secretory pathway, where they are secreted as proenzymes that must be activated by proteolysis, or (5) the mechanism of lysosomal exocytosis, where they are activated and thereafter secreted. Secreted proteases can bind to the cell surface through interference with other molecules, including CD44, integrins or (6) annexin II or through (7) tethering to ECM compounds. (8) Specific secreted proteases are attached to distinct receptors, for example, uPA couples to uPAR. (9) Proteases are capable to be liberated through exosomes originating from multivesicular bodies (MVBs), where they can be released into the extracellular cavity or into neighboring cells. The aggregation or the regional liberation of pericellular proteases are linked to microdomains on cell membranes, such as invadopodia, which are actin-rich moieties (10) or caveolae, which belong to “lipid raft” domains (11).

**FIGURE 3:**
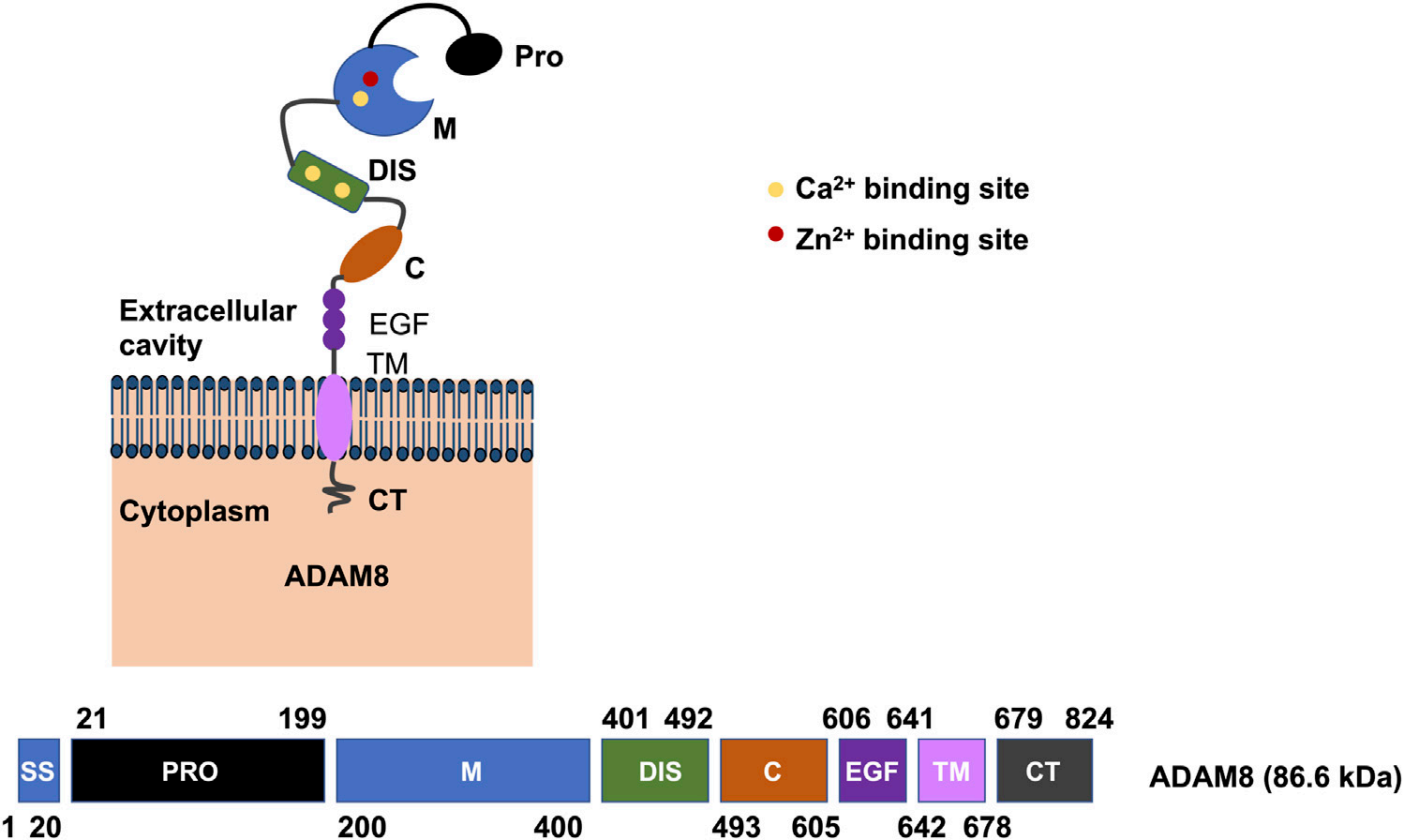
Illustration of the domains of human ADAM8. On the plasma membrane, the active human ADAM8 comprises a motif associated with ${Zn}^{2+}$ ions within the catalytic domain, that is, essential for the functionality (top image). There is also a motif for binding of a Ca^2+^ ion in the M domain and even two more in the DIS domain. Protein domains are schematically drawn, and amino acid positions are indicated below (bottom image). The domains are drawn in their sequential order by providing the amino acid numbers for the domains (bottom image). Domain name abbreviations are as follows: C, cysteine-rich; CT , cytoplasmic tail; DIS, disintegrin-like; EGF, epidermal growth factor-like; M, metalloproteinase; Pro, prodomain; SS, signal sequence and TM transmembrane.

ADAM8 is synthesized into a 120-kDa proform that can dimerize or multimerize and autocatalytically truncate its prodomain, thereby yielding an active membrane-associated metalloprotease of 90-kDa (Figure 2) (Schlomann et al., 2002). Active ADAM8 is capable of undergoing further processing by liberation of the MP domain into the ECM, retaining a membrane-associated 60-kDa residue form. ADAM8 cytoplasmic tail is quite elongated and features a conserved potential SH3 ligand domain, resembling that of ADAM9 (Weskamp et al., 1996). ADAM8 is composed of 824 aa and is considered a multidomain enzyme and consists of an N-terminal prodomain followed by a metalloproteinase (MP), a disintegrin (DIS), a cysteine-rich epidermal growth factor (EGF)-like transmembrane domain, and a cytoplasmic tail (CT) domain (Figure 3) (Yoshiyama et al., 1997). In particular, the prodomains could support folding of proteins engaged in protein synthesis and exert an autoinhibitory action on the catalytically active members of the ADAM family (Stone et al., 1999), thereby confirming that they are inactive in the early secretory route. Furin and furin-like proprotein convertase 7 (PC7) appear to be the two key enzymes that scissile the prodomains of ADAMs as they pass through the secretory route, thereby controlling their maturation and proteolytic activity. An exceptional feature of ADAM8 is its autocatalytic domain. More specifically, ADAM8 is capable of liberating its own prodomain through an autocatalytic mechanism (Schlomann et al., 2000). Curiously, an alternative autocatalytic activity has also been proposed for ADAM10 and ADAM17. However, this process does not involve the elimination of the prodomain and the activation of the protease under physiological conditions, but causes the degradation of the mature catalytically active types under experimental conditions in the course of the cell lysis process (Schlöndorff et al., 2000; Brummer et al., 2018). Consequently, ADAM8 is activated through autocatalysis in the trans-Golgi reticulum and needs the homophilic multimerization of at minimum two ADAM8 monomers at the cell membrane to become active *in vivo*. Notably, dimerization relies on the interference of the DIS-domains (Figure 2) (Schlomann et al., 2015). Proteases in endosomes or lysosomes are transported to the extracellular space through two different ways: firstly, the secretory pathway, in which they are liberated as proenzymes that need to be activated through proteolysis and secondly, through the lysosomal exocytosis, where they are activated and thereafter liberated (Figure 2). The regional liberation of the proteases is linked to microdomains, such as invadopodia and caveolae (Figure 2). In contrast to other family members like ADAM17 and ADAM10 (Mullooly et al., 2016), ADAM8 is non-essential in physiological states but has been upregulated in inflammatory processes and various cancers (Koller et al., 2009).

In the following, the knowledge about the different domains of ADAMs is briefly reviewed. The prodomain of ADAMs seems to serve two purposes: it inhibits activation of the zymogen and prevents intracellular transportation (Leonard et al., 2005). Similarly, in the instance of ADAM17, the prodomain is expected to inhibit its degradation while being transported toward the plasma membrane (Leonard et al., 2005). The metalloprotease domain comprises a conserved HEXXHXXGXXHD sequence, that is, necessary to perform the distinctive proteolytic activity (Figure 1). Even though all ADAMs possess the metalloprotease domain, as noted above, only about half of them exhibit protease activity (Table 2). All ADAMs incorporate a disintegrin domain that engages with integrins on neighboring cells and with ECM proteins (Reiss et al., 2006). Besides degrading ECM proteins and cleaving cell surface receptors, ADAMs are able to interact directly with other receptors such as integrins (Eto et al., 2000; Bax et al., 2004; McGinn et al., 2011; Conrad et al., 2019). The underlying structural determinants of the selectivity of disintegrin-integrin cross-talk are insufficiently characterized. In fact, based primarily on *in vitro* assays, it seems that multiple ADAMs can attach to the identical integrin, whereas specific integrins can commit to distinct ADAMs. The disintegrin domain of ADAMs is thought to play a role in cell adhesion and migration under physiological and pathological conditions through its binding to integrins. The cysteine rich domain is involved in the distinct substrate identification and controls the interplay between integrins and its disintegrin domain (Reddy et al., 2000). The majority of ADAMs consists of an epidermal growth factor (EGF)-like domain, that is, around 30–40 amino acids in size. EGF-like repeat sequences are conserved patterns in evolution that are present in many kinds of secreted and transmembrane proteins. These repeat sequences feature six cysteine residues that constitute three disulfide linkages. Certain EGF repeats carry consensus sequences for the accumulation of O-glycans (Haltom and Jafar-Nejad, 2015). The role of the EGF-like domain within ADAMs is ambiguous. The function of the transmembrane domain in ADAMs has been insufficiently explored. It is assumed that the function lies in the anchoring of these proteins to the plasma membrane (Haltom and Jafar-Nejad, 2015). The cytoplasmic or C-terminal domain differs in both length and sequence among members of the ADAM family. In ADAM10, the cytoplasmic domain has been shown to govern constitutive activity, while it is not being required for activity in response to stimulation (Maretzky et al., 2015). In ADAM17, phosphorylation of the cytoplasmic domain has been related to the mitigation of membrane disengagement through shedding (Díaz-Rodríguez et al., 2002).

Another characteristic feature of ADAM8 is that ADAM8 dimers can be consistently identified on the surface of ERα-negative breast cancer cells, whereas they are absent on ERα-positive breast cancer cells (Srinivasan et al., 2014). In human ADAM8, four N-glycosylation sites, such as Asn-67, Asn-91, Asn-436, and Asn-612 have been identified *via* site-directed mutagenesis. Prodomain sites Asn-67 and Asn-91 harbored abundant mannose, whereas complex type N-glycosylation was seen at Asn-436 and Asn-612 in the active and residual forms. In addition, ADAM8 N91Q and N612Q mutants show altered cellular localization and are incapable of targeting the cell surface. Thus, the Asn-91 and Asn-612 sites proved to be essential for proper processing and positioning on the cell surface, especially for their exit from the Golgi and the endoplasmic reticulum., respectively. The N436Q mutation resulted in reduced stability of ADAM8 because of increased lysosomal break down. Conversely, mutation of the Asn-67 site had only minor impact on the stability and processing of the enzyme. In conclusion, N-glycosylation is integral to the processing, localization, stability, and activity of ADAM8. Interestingly, while ADAM8 has been blocked by a range of peptide analog hydroxamate inhibitors, it has not been impaired by the TIMPs (tissue inhibitors of metalloproteinases) (Amour et al., 2002).

ADAM8 contains a multistructural operating domain and performs an essential function in extracellular proteolysis, including the liberation of chemokines and cytokines through ectodomain shedding, such as the immunomodulator, the low affinity IgE receptor CD23, TNF receptor 1 and IL-1 receptor 2, and cleavage of important ECM compounds, such as major constituents of the cancer stroma, such as collagen I, fibronectin and periostin (Conrad et al., 2019). With the disintegrin domain (DIS), ADAM8 can attach to integrins, such as the β1-integrin subunit, and thereby activate integrin signaling transduction pathways. The cytoplasmic domain is crucial for this activation and involves focal adhesion kinase (FAK), extracellular regulated kinase (ERK1/2), and protein kinase B (AKT/PKB) in signal transduction.

ADAM8 has been revealed to be a catalytically active protease (Table 2). Unlike other ADAMs, which depend on furin to shed the pro-domain and are thereby turned on, ADAM8 liberates its pro-domain though an autocatalytic process (Schlomann et al., 2002). ADAM8 is capable of splitting myelin basic protein and CD23 *in vitro*. Likewise, it cleaves peptides encompassing the prospective splitting sites of a variety of prototypical ADAM substrates, comprising Kit ligand-1 (KL1), TNFα, and amyloid precursor protein (APP) (Naus et al., 2006). The neuronal cell adhesion molecule “close homologue of L1” (CHL1) is also shed by ADAM8 as the enzyme and substrate are transfected together into COS7 cells, and thereafter conditioned culture medium harboring shed CHL1 initiates the growth of neurites, pointing to a designated involvement of ADAM8 in this process (Naus et al., 2004). In addition to its shedding potential, ADAM8 can also cleave ECM components, including fibronectin (Zack et al., 2009). Following pro-domain cleavage, a second autocatalytic process can liberate a soluble version of the catalytic domain of ADAM8, which is itself competent to disassemble the ECM (Zack et al., 2009). For proper biological function, ADAM8 needs to multimerize and tethers with β1 integrin on the cell surface (Zack et al., 2009). ADAM8 is associated with a role in neuroinflammation and thereby activates the NLRP3 inflammasome (Lu et al., 2021). ADAM8 has also been detected in endothelial cells during angiogenesis induction after tissue injury (Mahoney et al., 2009). In the following, the focus is on the function of ADAM8 in cancer and its malignant progression.

## 4 Role of ADAM8 in cancer

ECM elements can be altered in post-translational manner through a large number of secreted restructuring enzymes, including oxidases and proteases. While oxidases play a prominent role in primary solid cancers, cancer cell migration and metastasis (Liburkin-Dan et al., 2022), proteases are associated with malignancy progression because they have been localized prominently in the invadosomes, comprising podosomes and invadopodia, of migrating cells (Peláez et al., 2019; Maybee et al., 2022). In the majority of cancers, beyond the cellular portion, the non-cellular ECM environment in immediate proximity to a solid primary tumor is also markedly modified in its structure, composition, and molecules retained (Baghban et al., 2020) resulting in a change in the mechanical properties of the matrix (Watson et al., 2021). Thus, it can be hypothesized that ADAM8 is localized to invadosomes in a similar manner as specific MMPs.

**FIGURE 4:**
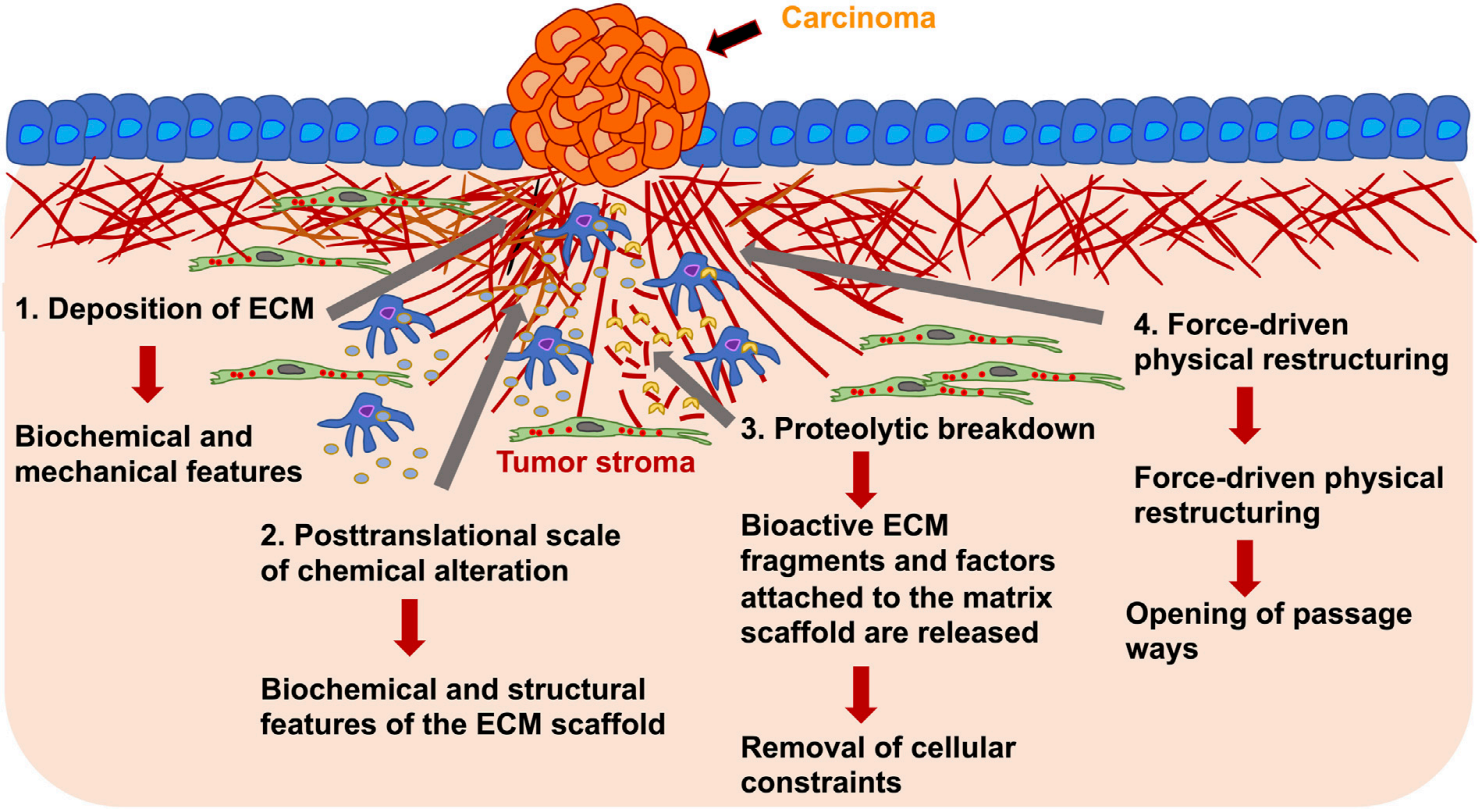
ECM remodeling of the tumor stroma around primary solid carcinoma occurs on various length scales. The remodeling of the tumor stroma can be caused by cancer cells and stroma embedded tumor associated cells, such as macrophages and fibroblasts. For example, there can be deposition of ECM molecules (1), posttranslational level of chemical alteration (2), proteolytic degradation through proteases, such as ADAM8 or MMPs (3) and force-driven physical restructuring (4). Through the ECM rearrangement are structural and mechanical characteristics affected that in turn alter the migratory capacity of cancer cells and may change the migration mode of cancer cells.

However, it arises a major question of what are the mechanisms of tumorigenic ECM remodeling. Alterations in the ECM, as seen in primary solid tumors, occur as a consequence of multiple remodeling mechanisms falling into four main processes: Firstly, the deposition of the ECM, that modifies the quantity and constitution of the ECM elements, thus impacting the biochemical and mechanical features of the ECM; secondly, posttranslational scale of chemical alteration, which modifies the biochemical characteristics and structural features of the ECM scaffold; thirdly, proteolytic break down, where bioactive ECM fragments and factors attached to the ECM are released and may be necessary for the removal of cellular confinements, such as migration barricades; and fourthly, force-driven physical restructuring, which influences the arrangement of the ECM through orienting the ECM fibers and facilitating the opening of passageways for cell migration (Figure 4) (Mouw et al., 2014; Yue, 2014).

Tissue homeostasis relies on the precise coordination between the abundance, modification, degradation, and restructuring of the ECM, which are reflected in the biochemical and biophysical characteristics of the ECM. Altering individual items can reverse the fragile equilibrium of ECM restructuring. Changes in the ECM have consequences for intricate cellular signaling distributions, as ECM constituents function as ligands for different cell surface receptors, like integrins, syndecans, and receptor tyrosine kinases. It is therefore not unexpected that cancer cells and tumor-associated stromal cells alter all four ECM remodeling mechanisms (Figure 4), resulting in a pro-cancer matrix that is an active participant in the pathology of the cancer (Kai et al., 2019). The aim of the following subparts is to review the supporting evidence for a specific function of ADAMs in cancer and to discuss the progress made in the development of anti-ADAM inhibitors for the therapeutic use of this disease.

The most important question that arises is whether there is a link between ADAM8 and malignant progression of cancer. Another follow-up question emerging is that of tumor specificity or that of a general mechanism independent of the tissue in which the tumor is localized. Although ADAMs are involved in numerous biological processes encompassing proteolysis, cell adhesion, cell fusion, cell proliferation, and cell migration, their involvement in proteolysis is the most widely explored. Only about half of the 22 ADAMs thought to be operational in humans, whereby only about half of them have MMP-like protease activity (Table 2). In contrary to MMPs, which are mainly responsible for the break down of ECM proteins, the major ADAM substrates comprise the ectodomains of type I and type II transmembrane proteins. Among them are growth factor/cytokine precursors, growth factor/cytokine receptors and cell-matrix or cell-cell adhesion proteins.

A high expression level of ADAM8 is strongly linked to a worse clinical outcome. For instance, ADAM8 expression is correlated with increased migration and invasiveness of PDAC cells caused through activation of ERK1/2 and increased MMP activities. In hepatocellular carcinoma (HCC), ADAM8 is highly expressed in murine HCC tissues and hepatoma cell lines, such as murine Hepa1-6 cells and human HepG2 cells (Awan et al., 2021). ADAM8 has been revealed as one of three potential tumor markers, whereby the others are LYN and S100A9, for metastasis and tumor reoccurrence in colon cancers (Liu et al., 2022). ADAM8 facilitated extracellular signal transmission in the local tumor environment of pancreatic cancer *via* the governed release of Lipocalin 2 and MMP-9 (Cook et al., 2022). In agreement, also other cancer types, such as breast cancer (Romagnoli et al., 2014; Conrad et al., 2018), lung cancer (Ishikawa et al., 2004), liver cancer (Zhang et al., 2013), pancreatic cancer (Valkovskaya et al., 2007), esophageal cancer (Chen et al., 2021), gastric cancer (Huang et al., 2015), glioblastoma (Dong et al., 2015; Schäfer et al., 2022) and colon cancer (Yang et al., 2014; Jin Q. et al., 2020) highlight a correlation between ADAM8 expression and cancer development and its malignant progression. When being upregulated in cancer, the proteolytic activity of ADAM8 is capable of fostering tumorigenesis through its ability to trigger angiogenesis and subsequently contribute to metastasis (Conrad et al., 2018). Indications of non-proteolytic roles of ADAM8 are also available, primarily attributable to the engagement of its DIS domain with β1-integrin on the cell surface. This type of two-way communication is considered to be important for the intracellular activation of the MAPK signaling transduction pathway, known to be implicated in the chemoresistance of cancer cells. When once upregulated, ADAM8 can interfere with the substrate spectrum of ADAM10 and ADAM17 and proteins with immune roles including TNF-R1 (Bartsch et al., 2010), L-Selectin (Gómez-Gaviro et al., 2007), CD23 (Fourie et al., 2003), and CXCL1 (Naus et al., 2006), as well as cell adhesion proteins, such as CHL1 (Naus et al., 2004), hereby possibly causing modulation of the immune response or cell adhesion.

Apart from the correlation of ADAM8 expression and the development and malignant progression of cancers, there is also a functional connection revealed. There are several techniques established to regulate the amount of ADAM8 in living cells, such as genetic knockdown, inhibitors or blocking antibodies. Last, but not least, the easiest way to alter the levels of ADAM8 is through the addition of the peptidomimetic ADAM8 inhibitor BK-1361. Specifically, the BK-1361 inhibitor has been designed through structural alteration of the disintegrin domain, and consequently impairs the multimerization of ADAM8. Inside PDAC cells, BK-1361 impairs the function of ADAM8 that causes impaired invasiveness, and diminished activation of ERK1/2 and MMP. In a mouse model system, BK-1361 reduced tumor load and metastasis of implant-derived pancreatic cancer cells. In addition, ADAM8 activity is hampered with BB94 (batimastat), GW280264, FC387, and FC143 (two ADAM17 inhibitors) and attenuated with GM6001, TAPI2, and BB2516 (marimastat), whereas no impairment has been seen for GI254023, which has been reported to be an ADAM10-specific inhibitor (Schlomann et al., 2019).

To investigate the probable involvement of ADAM8 in the tumor microenvironment, pancreatic ductal adenocarcinoma (PDAC) cancer cells, such as Panc89 and AsPC1 cells, have been an engineered CRISPR/Cas9 knockout of ADAM8 and their liberation of extracellular vesicles has been scrutinized. In extracellular vesicles, ADAM8 is abundant as an active protease and can be linked to lipocalin 2 (LCN2) and MMP-9 in an ADAM8-dependent fashion, as ADAM8-KO cells exhibit a reduced frequency of LCN2 and MMP-9. ADAM8 sorting occurs independently of TSG101, a protein, that is, part of the ESCRT pathway, despite the fact that ADAM8 contains the recognition motif PTAP for TSG101 in its CD domain (Cook et al., 2022). In agreement, CRISPR/Cas9 knockout of ADAM8 in human breast carcinoma cells resulted in the same results (Cook et al., 2022), which indicates that the phenomenon of ADAM8 is not limited to a specific cancer type.

Consequently, ADAM8 level has been tightly linked to the invasion and metastasis of several malignant cancers and affected the overall outcome of cancer patients’ prognosis. The five mechanisms involved in the progression of cancer by ADAM8 are (Jin Q. et al., 2020): firstly, the creation of a tumor-friendly microenvironment; secondly, the disassociation of ECM compounds; thirdly, generation of chemical opposition and regulation of extracellular activity of MMPs; fourthly governance of cell motility and adhesion and fifthly, triggering the generation of new blood vessels. Beyond ADAM8’s function in cancer, it is coupled inflammation (Koller et al., 2009), which may also play a role in advancement of cancer. Finally, it can be hypothesized that ADAM8 is involved not only in the malignant EMT transition of colon cancer, but also of other cancers at the doorstep of the transition.

## 5 Epithelial-mesenchymal transition (EMT) of cancers

Epithelial-mesenchymal transition (EMT) involves a biological process in which epithelial cells cease to be epithelial and take on mesenchymal characteristics. Throughout EMT, epithelial cells experience deprivation of cell-cell junctions, apical-basal polarity, and epithelial biomarkers, and become more motile, adopt a spindle-like appearance, and attain mesenchymal markers (Kalluri and Weinberg, 2009). These morphological alterations are accompanied by a suppression of epithelial markers, including E-cadherin, claudins and occludins, and an increase in mesenchymal markers, including vimentin, fibronectin and N-cadherin (Nieto, 2011; Lamouille et al., 2014; Nieto et al., 2016; Yang et al., 2020). Originally, the concept of EMT has been formulated as epithelial-mesenchymal transformation by Elizabeth Hay in 1968 (Hay, 2005) to refer to the key cellular alterations in embryogenesis; it was afterwards retitled as EMT to discriminate it from neoplastic transformation (Nieto et al., 2016; Yang et al., 2020). EMT and its inverted counterpart process, mesenchymal-epithelial transition (MET), reveal fundamentals in several physiological and pathological events. Originally, EMT has been inferred to be a process vital for the generation of the body structure and various tissues and organs throughout embryonic development, such as the building of somites and the development of the heart (Thiery et al., 2009). Subsequently, EMT has been determined to play a role in adult organisms, under strict regulation and very specific conditions (Thiery et al., 2009; Stone et al., 2016). For instance, EMT is triggered after an injury to induce re-epithelialization and restructuring of the ECM in the course of the wound healing event (Thiery et al., 2009; Lim and Thiery, 2012; Nieto et al., 2016).

EMT is also implicated in cancer advancement and tissue fibrosis (Nieto et al., 2016; Dongre and Weinberg, 2019; Pastushenko and Blanpain, 2019; Williams et al., 2019). EMT and MET are prevalent in a variety of biological settings and exhibit highly plastic and dynamic performance across cell fate transitions. In specific, the process of metastasis is typified by the depletion of the characteristics of epithelial cells and the adaption of the features of mesenchymal cells (Tiwari et al., 2012; Brabletz et al., 2018; Mitchel et al., 2020; Ribatti et al., 2020). EMT involves the transition, whereby epithelial cells acquire the capacity to invade, withstand stress, and spread (Hanahan and Weinberg, 2011; Saxena et al., 2020). EMT is governed at multiple scales through numerous determinants, encompassing cell signaling, transcriptional guidance, epigenetic alteration, and post-translational modifications. Epithelial cells sustain EMT-inducing cues from their niches. For instance, cytokines inducing the transforming growth factor-β (TGF-β), hepatocyte growth factor (HGF), epidermal growth factor (EGF), and fibroblast growth factor (FGF) families can trigger or stimulate the EMT event (Zavadil and Böttinger, 2005; Thiery and Sleeman, 2006; Nieto et al., 2016; Williams et al., 2019).

These EMT-trigger cues upregulate distinct transcription factors (referred to as EMT-TFs) of the SNAIL, TWIST, and ZEB family (Thiery and Sleeman, 2006; Valastyan and Weinberg, 2011; Nieto et al., 2016; Williams et al., 2019; Park et al., 2022). EMT inducing transcription factors (EMT-TFs) typically partner with miRNAs and epigenetic and/or post-translational regulators to direct EMTs (Ye and Weinberg, 2015; Nieto et al., 2016). For instance, the miR-200 family, comprising miR-141, miR-200a, miR-200b, miR-200c, and miR-429 fulfils a critical function in repressing EMT through impairment of the translation of ZEB1 and ZEB2 (Korpal et al., 2008; Park et al., 2008). In cancer, the EMT program endows these epithelial cells with characteristics that are pivotal for invasion and metastatic spread, such as enhanced motility, invasiveness, and the capacity to break down constituents of the ECM (Nieto et al., 2016). These complex metastatic staging processes are coordinated through a set of EMT-TFs that have been comprehensively explored (Craene and Berx, 2013; Lamouille et al., 2014).

Perhaps most difficult in the investigation of EMT is that the transitions between epithelial and mesenchymal states are not a binary process. In their place, cancer cells frequently harbor a range of epithelial and mesenchymal features (Jolly et al., 2015, 2017; Zhang and Weinberg, 2018). E-cadherin, occludins, and cytokeratins represent the most frequently employed markers for epithelial characteristics, while N-cadherin and vimentin are the most widely accepted markers for the mesenchymal condition (Thiery et al., 2009). Several investigations have revealed that distinct cancer cells, comprising breast, pancreatic, renal, lung, and colorectal cancers, express both epithelial and mesenchymal markers (Bronsert et al., 2014; Sampson et al., 2014; Zhang et al., 2014; Schliekelman et al., 2015; Hiew et al., 2018). Consequently, there also the possibility that the EMT of numerous cancer cells may be not a complete EMT, instead, these cells display intermediate, or hybrid epithelial/mesenchymal E/M phenotypes. The partial EMT or hybrid E/M phenotype have several benefits compared to the full EMT phenotype, including ease and speed of adaptation to environmental signals. This characteristic feature is referred to as epithelial-mesenchymal plasticity (EMP) (Ye and Weinberg, 2015).

Epigenetic and post-translational regulators equally have a decisive part to play in the supervision of the EMT process (Nieto et al., 2016). However, for a long time not much was known about the interplay between ADAM8 and EMT, although it has been hypothesized that ADAM8 is critical for cancer invasion and metastasis. This has changed due to recent studies. Analysis of human colon cancer data from TCGA and GTEx databases identified that ADAM8 is strongly linked to adverse prognosis in patients suffering from colon cancer, but also identified a relationship between ADAM8 and EMT-related biomarkers. Moreover, ADAM8 has been detected to correlate with the expression of multiple EMT biomarkers, comprising E-cadherin, N-cadherin, Vimentin, Snail2 and ZEB2 (Jin P. et al., 2020). Besides, ADAM8 is able to trigger EMT to enhance colon cancer cell penetration through activation of the TGF-β/Smad2/3 regulatory signal transduction pathway (Jin Q. et al., 2020). Thus, these findings seem to be promising that ADAM8 fulfills crucial task in regulation of EMT, possibly also through its interaction with miRNAs (Das et al., 2016).

ADAM8^+^ immune cells are able to extravasate through endothelial linings of blood vessels and invade the ECM, as it has been reported for specific inflammation models (Naus et al., 2010; Schick et al., 2019). Based on these outcome, it can be speculated that ADAM8 could also perform a functional task within immune cells of the tumor microenvironment (Cook et al., 2022).

## 6 The effect of ADAM8 on cell motility and co-culture

### 6.1 ADAM8’s function in cell motility

ADAM8 has been implicated in the increase of cellular motility in pancreatic cancer (Valkovskaya et al., 2007), in breast cancer (Das et al., 2016), in primary brain cancer (Wildeboer et al., 2006) and prostate cancer (Fritzsche et al., 2006). For instance, in breast cancer cells, it has been revealed that ADAM8 regulates specific miRNAs, such as miR720 (Das et al., 2016). Silencing of ADAM8 in human MDA-MB-231 breast cancer cells reduced their migratory potential to invade Matrigel (Das et al., 2016). ADAM8 enhances early metastatic events including transendothelial migration through upregulation of MMP-9 and liberation of PSGL-1 from breast cancer cells. The MMP-9 function may depend on the cancer disease state, such as the presence of fibronectin, which in turn elevates the level of proteases. To examine the role of ADAM8 in metastasis, MDA-MB-231 breast cancer cells with ADAM8 knockdown, named MB-231_shA8, and scramble control cells, named MB-231_shCtrl, have been examined for their capacity to form metastases. *In vitro*, the assembly of metastatic complexes in suspended droplets (hanging drops) relies on ADAM8 and is prevented by ADAM8 blockade. MB-231_shA8 cells, unlike MB-231_shCtrl cells, were compromised in transmigration through an endothelial and a remodeled blood-brain barrier. Among the 23 MMP and 22 ADAM genes, it was only the MMP-9 gene that could be impacted by silencing of ADAM8 in MB-231_shA8 cells. Reexpression of wild-type ADAM8, as opposed to ADAM8 missing the cytoplasmic domain, in MB-231_shA8 cells led to enhanced values of activated pERK1/2 and pCREB (S133), which were accompanied by enhanced MMP-9 transcription. Several phases of the metastatic staging chain demand proteolytic activity (Mason and Joyce, 2011). Mechanistically, in addition to matrix restructuring and break down, proteases also shed adhesion molecules, release growth factors, and activate kinases (Murphy, 2008; López-Otín and Hunter, 2010; Mason and Joyce, 2011). Circulating disseminated cancer cells access the destination organ by mechanisms similar to the rolling of leukocytes on the vascular wall luminal surface, which is promoted on the endothelial side through a class of carbohydrate-binding proteins including E− and P-selectins of which the ligands are ESL-1, CD44, and PSGL-1 (McEver and Cummings, 1997; Ley et al., 2007). In a manner analogous to leukocytes, circulating cancer cells also express these selectin ligands upon their cell surface (Läubli and Borsig, 2010). Further posttranslational processing of these ligands on the cell membrane surface, as carried out through sheddases (Zarbock and Rossaint, 2011), governs the equilibrium between adhesion and motility of circulating cancer cells. After attachment, intraluminal creep, and ultimately firm adherence to the endothelial lining, endothelium-bound cancer cells are able to proliferate and establish intravascular cancer microcolonies as metastatic foci (Al-Mehdi et al., 2000) or they undergo extravasation *via* necroptosis of endothelial cells through APP and DR6 (Strilic et al., 2016).

As mentioned before shedding comprises enzymes, which are members of the metzincin superfamily, such as MMPs, MT-MMPs and ADAMs. Several MMPs and members of the ADAM protease family have been delineated for proteolytic treatment of the aforementioned ligands. ESL-1 molecules are broken down through a yet unknown metalloprotease, and the liberation of ESL-1 in microglial cells and macrophages is triggered through lipopolysaccharide (Werneburg et al., 2016). The hyaluronic acid receptor CD44 can be split through MT-MMP1 (Terawaki et al., 2015) and by ADAM10 (Hartman et al., 2016). The P-selectin ligand PSGL-1 is broken down by ADAM8. This has been first shown in zebrafish (*Danio rerio*), where deficiency of ADAM8 resulted in a retardation of the commencement of blood circulation, which is attributable to the strong attachment of leukocytes to endothelial cells of the vascular system (Iida et al., 2010). Consequently, the key impact evoked through the shedding of selectin ligands is an elevation in motility and invasiveness into targeted tissues or epithelia (Murphy, 2009). Interaction with cellular integrins allows ADAM proteases to agglomerate in cellular protuberances and convey through active proteolysis the barrier effect (Bax et al., 2004). Moreover, integrin-facilitated signal transduction pathways can augment the extracellular activities of MMPs, such as the increase of fibronectin can upregulate the expression of MMP-2 and MMP-9 in human MCF-7 breast cancer cells (Das et al., 2008). MMPs additionally drive the loosing of basement membrane intactness and permit direct invasion of carcinoma cells due to proteolytic break down of tight junctions and ECM proteins (Voura et al., 2013; Rempe et al., 2016). Related to this, MMP-9 facilitates transendothelial migration (Rempe et al., 2016) and it is probable that co-regulation of MMP-9 with de-adhesion of cancer cells to the endothelium is integral to the metastatic progression. Lately, the metalloprotease disintegrin ADAM8 has been characterized in the association with PSGL-1 shedding (Domínguez-Luis et al., 2011) and the spread of breast cancer cells (Romagnoli et al., 2014). A high expression rate of ADAM8 is accompanied by an augmented amount of circulating cancer cells and subsequently a higher abundance of brain metastases in orthotopic mouse models (Romagnoli et al., 2014).

The expression of ADAM8 in brain metastases from various primary malignancies has been investigated, and ADAM8 has been found to be elevated in brain metastases derived from primary breast cancer and to aid transmigration across the endothelium and blood-brain barriers. Evidence has also been provided that the observed proteolytic action of ADAM8 is attributable to MMP-9 expression and activity upregulation. Mechanistic evidence for regulation of MMP-9 expression by ADAM8-driven signal transduction in breast cancer cells is presented, lending weight to the idea that metalloproteases can mutually regulate one another in cross-talks collectively referred to as the “protease web” (Overall and Dean, 2006).

The use of ADAM8 and MMP-9 antibodies diminished the transmigration of MDA-MB-231 cells, indicating that ADAM8 impacts the levels of transmigration of breast cancer cells by regulating MMP-9. ADAM8-driven transmigration has been validated in Hs578t cells that overexpress ADAM8. Additionally, transmigration of MDA-MB-231 and Hs578t cells has been markedly attenuated when the cells were exposed to an antibody directed against PSGL-1, which is a substrate of ADAM8. Based on these datasets, it can be deduced that ADAM8 enhances precocious metastatic events like transendothelial migration through upregulation of MMP-9 and liberation of PSGL-1 by breast cancer cells. Finally, the effect of ADAM8 on the transmigration of cancer cells is not restricted to a single cancer type, it seems to be a rather frequent or general phenomenon.

### 6.2 ADAM8 in co-culture model systems

When cancer cells are co-cultured with macrophages (THP-1 cells), the expression of LCN2 and MMP-9 has been found to be induced in ADAM8-KO cells, indicating that macrophage signal transduction can override ADAM8-driven intracellular signal transduction in PDAC cells. When co-cultured with macrophages, the regulatory effect of MMP-9 is found to be unaffected from the M1/M2 polarization state while the LCN2 expression is influenced preferably by M1-like macrophages. Based on these results, however, molecular data suggest that ADAM8 has a systemic effect in the tumor microenvironment and that its expression in different cell types needs to be taken into account for the specific treatment ADAM8 in cancers (Cook et al., 2022). Additionally, it needs to be considered that ADAM8 is present in extracellular vesicles, where is it stored as an active protease. First and foremost it has been established that the substantial functioning of ADAM8 lies in the extracellular liberation of MMP-9 and LCN2, which are two key facilitators of cancer advancement in PDAC (Cook et al., 2022).

A severe desmoplastic stromal reaction to cancer growth is a hallmark of PDAC and, in part at least, a reason for the disastrous overall prognosis of patients (Huber et al., 2020). Specifically, the tumor microenvironment with its inflammatory character activates multiple immune cell types and represses the immunocompetence of the tumor microenvironment, indicating extensive tumor-immune cell exchange through the ECM.

ADAM proteases, acting as membrane shedding enzymes, are competent to generate intercellular cues through the controlled liberation of membrane proteins implicated in immunomodulation. Such an ADAM protease is ADAM8, which has been proven to exhibit tumor-supportive activities when expressed in cancer cells, thereby promoting cancer progression, invasion, and enhancing immune cell enrolment (Schlomann et al., 2015; Conrad et al., 2019). Most recently, a more systematic dissection of tumor-associated immune cells in cancer tissue derived from PDAC patients identified that ADAM8 is also expressed in macrophages, neutrophils, and NK cells (Jaworek et al., 2021). These findings are in agreement to experiments, in which ADAM8 first appeared in macrophages and macrophage-like cell lines after screening a cDNA library containing genes upregulated in response to lipopolysaccharides (LPS) (Yoshida et al., 1990).

ADAM8^+^ immune cells are able to traverse endothelia and penetrate the ECM, as evidenced in several inflammatory models (Naus et al., 2006; Schick et al., 2019). These results imply that ADAM8 may also exercise its proper function in immune cells of the microenvironment of primary solid tumors. In view of the high levels of endogenous expression of ADAM8 in macrophages, it has been hypothesized that ADAM8 performs essential functions in the interactions between cancer cells and macrophages. These interactions potentially might be conveyed through extracellular vesicles, which is a particular type of lipid-enclosed particle with a size varying from 30 to 100 nm. ADAM8 has been characterized as an extracellular vesicle load and has diagnostic power for the screening of PDAC lesions (Verel-Yilmaz et al., 2021). Finally, the interplay between cancer cells and immune cells needs to be explored in a more detailed manner. Thereby, the exact roles of the two cell types and the expression of ADAM8 have to be identified in terms of alteration cell mechanical or matrix mechanical phenotypes. In general, the importance of ADAM8 in cancer progression is misjudged and it has not been subject of many cancer studies, although it has the power to act as a key molecule in various timepoints in cancer evolution and its malignant progression.

## 7 ADAM8 is involved in regulation of matrix remodeling and mechanical fiber displacement

Collagen is the main component of the ECM environment in vastly all kinds of tissues, and its content is altered in the microenvironment of tumors and also inflammatory diseases. Collagen is thus a perfectly suitable object for studying the restructuring of the cellular environment by ADAM8. Through cell-based restructuring of the collagen matrix, communication with other cells in the cancer stroma can be established. Moreover, collagen and/or a collagen matrix could be a tool to codify disease effects and/or mechanical alterations.

The role of ADAM8 has been increasingly recognized in cancer research, with a focus on malignant progression that involves restructuring the environment of primary tumors. In particular, MMP-9 expression has been found to be increased in ADAM8-positive cancers. As a collagenase MMP-9 is assumed to function as a crucial degradative enzyme in the scheme of ECM degradation. Moreover, secreted MMP-9 is capable to localize to the plasma membrane through tethering to ανβ3 (Rolli et al., 2003) and α4β1 integrins (Redondo-Muñoz et al., 2008) or CD44 (Yu and Stamenkovic, 1999). In the past, the major collagenases comprising MMP-1, MMP-9, MMP-13, and MMP-14 (synonymously referred to as MT1-MMP) have been identified to break down collagens and collagen matrices (Rose and Kooyman, 2016). Consequently, ADAM8 fulfills a matrix remodeling function through its regulation of metalloproteinases, such as MMP-9.

**FIGURE 5:**
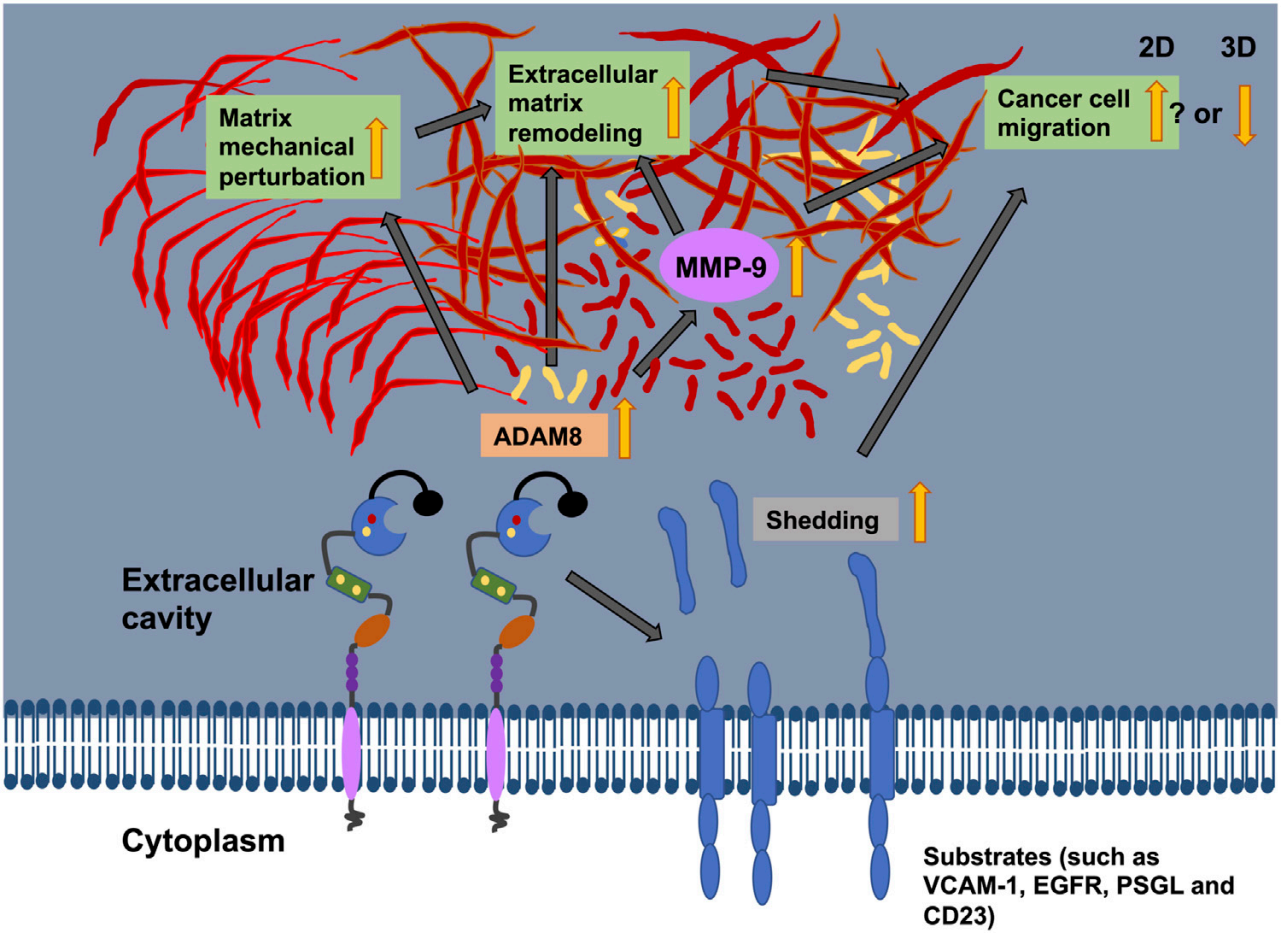
The illustration shows the potential relationship between ADAM8 and cancer cell migration, matrix mechanics and ECM remodeling. ADAM8 can shed cell surface receptors, such as VCAM-1 CD23, PSGL, and EGFR, from the plasma membrane surface. Thereby, the cell adhesion and cell migration behaviors can be altered. ADAM8 can also cause an upregulation of the expression of MMP-9 that leads to elevated proteins levels and subsequently ECM degradation (indirect way). ADAM8 can also break down the ECM (direct way). Recently, ADAM8 can perturb the mechanical characteristics of the matrix through reduced fiber displacements. All these functional impacts of ADAM8 on cancer cells can impact cancer cell motility in 2D and 3D environments, however, the motility seems to be elevated in 2D systems, whereas the motility impaired in 3D ECM hydrogels.

MMPs are historically named matrixins and are a family of matrix remodeling and degrading enzymes. The ECM is composed of hundreds of molecules, among them proteoglycans, glycosaminoglycans, structural proteins, like collagen and elastin, adhesion proteins, such a fibronectin and laminin, and proteases such as MMPs (Cui et al., 2017). There are 24 MMP genes in humans that encode for 23 different MMPs, whereby the MMP23 is encoded by two identical genes. All MMPs possess typically a N-terminal secretory signal peptide domain, a propeptide with around 80 amino acids with a conserved PRCGXPD motif, a catalytic metalloproteinase domain with around 170 amino acids, a linker peptide (hinge region) with variable size, and a hemopexin domain with around 200 amino acids (Cui et al., 2017). The traditional way to subgroup MMPs is based on substrate specificity, sequence similarity and structural domain architecture. Consequently, there are the following subgroups of MMPs: Collagenases, gelatinases, stromelysins, matrilysins, membrane-type MMPs, and other MMPs (Cui et al., 2017). A new bioinformatics-based analysis of MMPs an divide them into five distinct types (Cerofolini et al., 2019): first type are non-furin regulated MMPs comprising MMP-1, -3, -7, -8, -10, -12, -13, -20, and -27, second type are MMPs containing three fibronectin-like parts within the catalytic domain, including MMP-2 and -9; third type are MMPs coupled to the cellular membrane through a C-terminal glycosylphosphatidylinositol (GPI) moiety, comprising MMP-11, -17 and -25; fourth type cover MMPs possessing a transmembrane domain, including MMP-14, -15, -16, and -24) and the fifth type harbor all other MMPs, encompassing MMP-19, -21, -23, -26, and -28. The regulation of MMP-9 by ADAM8 is an indirect way to break down and restructure the ECM through the cleavage of fibrillar collagen (Figure 5). However, there exists also a direct way through cleavage of collagen by ADAM8’s proteolytic activity (Hayn et al., 2023).

Apart from the remodeling of collagen, ADAM8 can cleave fibronectin (Figure 5) (Zack et al., 2009). The modulation of fibronectin and its degree of fibrillarization within the ECM is critical for the progression of cancer disease (Libring et al., 2020). Moreover, the glycoprotein fibronectin is of particular concern because fibronectin expression in primary breast cancer is highly associated with poorer patient survival in all mammary cancer subtypes (Balanis et al., 2013).

Even in ADAM8 expressing cells, fibronectin is capable to induce the expression of MMP-9 (Figure 5) (Sen et al., 2010). This action seems to be primarily imparted through the integrin receptor α5β1, since inhibition of α5 abolished the fibronectin-facilitated excitatory responsiveness to MMP-9. Consequently, MMP-9 breaks down fibronectin. Among all MMPs, MMP-9 has been shown to have a critical function in the growth and metastasis of cancer cells. MMP-9 cleaved fibronectin can bind to αvβ6 integrins and foster the migration of breast cancer cells (Figure 5) (Li et al., 2015). Consequently, this is associated with a poor prognosis of cancer. Apart from the indirect degradation of fibronectin there is also a direct break down of fibronectin through ADAM8. Similar to other members of the ADAM family, ADAM8 is also recognized for proteolytic digestion of membrane-bound precursors (shedding activity) and alteration of cell-cell and cell-matrix interfaces. ADAM8 can directly break down fibronectin in the VRAA271 neoepitope (Zack et al., 2009). All of which may foster the migration and invasion of cancer cells, which utilize an amoeboid migration mode and consequently the malignant progression of cancer.

Based on these findings, it can be hypothesized that ADAM8 is predisposed to sense the mechanical cues from the ECM environment and possibly interferes with it in a mechanical manner. Recently, ADAM8 has been reported to displace collagen fiber networks and more severely also fibronectin-collagen matrices, which has been determined using 3D fiber displacement analysis (Hayn et al., 2023). Human MDA-MB-231 breast carcinoma cells with ADAM8 knocked down, referred to as ADAM8-KD cells, and scrambled control (ADAM8-Ctrl) cells have been employed to explore cell migration in 3D collagen matrices. Unexpectedly, it has been shown that ADAM8-KD cells migrated more numerous and deeper in 3D collagen matrices compared to ADAM8-Ctrl cells (Hayn et al., 2023). Moreover, it has been revealed that ADAM8-KD cells displaced collagen fibers significantly more than ADAM8-Ctrl cells. The elevated migratory capacity can be attributed to increased fiber displacements. Impairment of ADAM8 with ADAM8-inhibitor BK-1361 in ADAM8-Crtl cells led to similar results, since the fiber displacements of ADAM8-Ctrl cells were raised to the levels of ADAM8-KD cells (Hayn et al., 2023).

## 8 Final remarks and future perspectives

Identification of specific new molecular biomarkers and comprehension of emerging mechanisms implicated in the advancement of metastatic cancer are required for an earlier detection and improved treatments. Thereby, ADAM8 has been found to perform a key role in the progression of malignant cancer, that is, not restricted to a single cancer cell type but seems to be a fairly universal phenomenon, as it has been noticed in colorectal cancer (Jin P. et al., 2020), glioblastoma (Schäfer et al., 2022), breast cancer (Romagnoli et al., 2014; Conrad et al., 2018), hepatic cancer (Awan et al., 2021), pancreatic cancer (Schlomann et al., 2015), lung cancer (Ishikawa et al., 2004), brain cancer (Li et al., 2021) and gastric cancer types (Chung et al., 2019). The detection of ADAM8 in almost all cancers and especially in those with malignant progression implies a universal role of ADAM8 in cancer and its advancement. Moreover, ADAM8 has been identified to serve a functional role, as impairment of ADAM8 led to a better prognosis in several cancer types (Romagnoli et al., 2014). Since ADAM8 is even not restricted to cancer cells, it cannot simply be inhibited by specific drugs, when these drugs are not embedded into cell-type specific delivery systems. Moreover, the timing of the drug delivering may be crucial for the success of cancer treatment and consequently for the overall outcome. Therefore, a better comprehension of the role of ADAM8 in tumor microenvironments, such as ECM remodeling and stroma cells is critical. As ADAM8 is a proteolytic enzyme, ECM remodeling occurs and hence, the mechanical characteristics of the microenvironment may also change. Future efforts are needed to explore the role of ADAM8 in 3D ECM remodeling, investigating not only biological functions but also physical functions. In particular, the composition and structural orientation of fibers within the ECM scaffold appear to be of potential interest for the proper functioning of ADAM8-driven matrix remodeling and proteolytic protein cleavage. Apart from this, also mechanical properties of the ECMs may play a role. In addition, matrix-embedded cells, such as immune cells, may also facilitate or hinder the migration of a particular cancer cell type, which must be given special consideration because ADAM8 expression has also been observed in tumor stromal cells, such as macrophages. Alteration of cell-matrix and/or cell-cell adhesion, detachment of membrane proteins, and cleavage of ECM proteins and subsequent release of stored growth factors, cytokines, or truncated proteins can all contribute to altering the mechanical properties of the tumor stroma. These mechanical changes of the matrix in turn may affect the mechanophenotype of the cancer cells and possibly also other embedded cells, which subsequently may also alter their functions. The mechanical alteration of the cells has to be elucidated, and as a consequence, the entire mechanotransduction process needs to be clarified in a more detailed way. As there is an interaction between integrins and ADAM8, this connection has to be analyzed in terms of altered force generation and transmission. Since ADAM8 appears to play a role in the displacement of fibers from the ECM environment, the connection of ADAM8 to the three major components of the cytoskeleton, such as microfilaments, intermediate filaments, and microtubules, is a future topic of investigation. The functions of ADAM8 appear to be relatively universal across a broad spectrum of cancers, supporting the controversial hypothesis that there are specific general mechanical mechanisms, such as the mechanical properties of cancer cells, that share a common characteristic across a broad spectrum of cancers.
